# Shortage in *X*-Ray Operators

**Published:** 1915-08-28

**Authors:** 


### SOME RESULTS OF WAR TIME.

### Shortage in X-Ray Operators.

To those who have a close knowledge of hospital conditions at the present time it might appear that the great pains that were taken at an inquest at Preston Royal Infirmary on August 20 to remove any possible misapprehension that an apparent delay in the treatment of a case had anything to do with the patient's death were quite superfluous. But not everybody, even in these days, is fully able to appreciate the difficulties that confront hospital administrators and medical staffs, and for this reason the expressions of the Coroner, the jury, and the patient's nearest relative were not only satisfactory but very necessary. The case was one in which an eleven-year-old boy, who was pretending to do some conjuring tricks for the benefit of his younger sister, had the misfortune to swallow a penny. The coin lodged tightly in the boy's throat, and he was removed to the Chorley Hospital, where (according to the statement of the father) the *x*-ray apparatus did not seem to be powerful enough to locate the position of the obstruction. The boy was taken to Preston Infirmary in a taxi-cab, and on the following day the penny was removed. The evidence of Dr. J. B. Tindall, honorary medical officer, was that no injury was done to the throat by the operation of removing the penny. Septic inflammation of the lungs developed two days later, and death ensued. The septic condition was directly caused by the injury done to the throat by the penny, and not by any subsequent treatment. The boy's father said that when he arrived at the infirmary there seemed to be nobody at the hospital to manage the *x*-rays, and Dr. Tindall said the staff were short-handed at present on account of the war. All the available *x*-ray operators had been taken for war work, and it was only possible to get competent operators from outside on certain days. Answering the Coroner (Mr. J. Parker), Dr. Tindall said he did not think it would have made any difference if the penny had been removed on the day the boy was brought, as the damage to the throat had already been done. The boy's father expressed his gratefulness to the infirmary staff for their work, but added that he thought possibly the risk of poisoning might have been reduced if the obstruction could have been removed earlier. The Coroner pointed to the dearth of skilled operators, and to the extremely valuable voluntary service which is being rendered daily by Dr. Tindall and other "volunteers," and pointed out that it was clear everything had been done that could be done, and that the same result would have occurred had the penny been removed earlier. The jury, in returning a verdict of "Death from misadventure," expressed the view that the staff of the infirmary had done everything they possibly could do. The whole incident is lamentable.

